# Is breastfeeding ‘exclusive’? Barriers facing global health professionals and proposed solutions

**DOI:** 10.1371/journal.pgph.0004377

**Published:** 2025-03-26

**Authors:** Meike J. Schleiff, Devaki Nambiar, Frehiwot Nigatu Yimer, Natalia Ilona Varallyay, Sarah L. Dalglish

**Affiliations:** 1 Global Business School for Health, University College London, London, United Kingdom; 2 The George Institute for Global Health, New Delhi, India; 3 Project HOPE, Addis Ababa, Ethiopia; 4 Independent Global Health Consultant, Buenos Aires, Argentina; 5 Institute for Global Health, University College London, London, United Kingdom; PLOS: Public Library of Science, UNITED STATES OF AMERICA

Global public health champions breastfeeding and sets global norms recommending that women breastfeed exclusively on demand for six months and continue up to two years and beyond [1]. Yet in our experience, it is difficult to meet these norms while working as global health professionals, let alone pursuing career advancement and leadership roles for which women are already in the minority [2]. This situation must change: solutions are available. Without them women are professionally disadvantaged and excluded from making their highest potential impact.

Despite substantial privilege, we have struggled to breastfeed while working in diverse global health settings including international organisations, academia and civil society organizations around the world. While we come from different geographic regions – Africa, south Asia, Europe, and North and South America – each with varying cultures and laws, we have all faced challenges including unsanitary conditions, lack of infrastructure, insensitive managers and colleagues, and unyielding workloads that did not accommodate the hours of daily labor required to breastfeed, chestfeed or express milk. We believe these experiences are shared by many; constructive and frank conversation and remedial actions at organisational and systemic levels are needed.

The evidence is clear that human breastmilk is an excellent source of nourishment (and comfort) for babies, with the field of global health making great strides to highlight these benefits [3]. However, rates of breastfeeding remain sub-optimally low, with only 37% of infants breastfed in low- and middle-income countries [4]. In diverse contexts, women experience the return to work as a difficult moment for continued breastfeeding, citing physical and emotional difficulties, lack of appropriate facilities and lack of social support [5,6].

Most working women cannot be with their babies for every feeding, leading to an entire industry related to expressing and storing milk, which facilitates breastfeeding while also being costly financially and in terms of time. The ‘right to pump’ has been established [7], and in some cases is being implemented in a purpose- and values-driven fashion - yet this is far from a universal norm. Women returning to work after giving birth note the lack of a suitable environment to express milk as a significant barrier to continuing breastfeeding [8].

Furthermore, breastfeeding today frequently has negative consequences on women’s participation in the workforce and leadership possibilities, including in global health, which often requires substantial travel to support partnerships and deliver on programmatic and organizational goals [9]. Educated women in professional and/or managerial positions, who frequently have the highest rates of both breastfeeding initiation and return to work after maternity leave, nonetheless also often experience continued breastfeeding as “taboo” and are obliged to either stop breastfeeding or hide breastfeeding-related activities [10]. Indeed, we have experienced discomfort around the practical aspects of continued breastfeeding despite global health’s nominal embrace of it.

We outline a list of barriers and burdens we have collectively faced while breastfeeding as well as actionable recommendations for diverse global health organisations that would alleviate these issues (visualized in Fig 1 and further described in S1 Table). We recognize that global health is a diverse field, and so we have likely not addressed every challenge.

**Fig 1:**
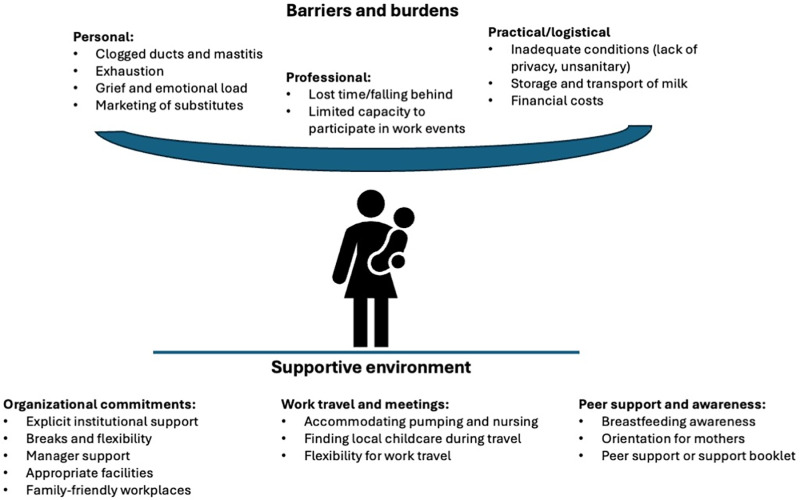
The barriers and burdens on breastfeeding women and the role of a supportive environment to overcome them.

WHO guidance provides recommendations for supporting breastfeeding women returning to work in its *Global Strategy for Infant and Young Child Feeding*, including paid work breaks and the provision of facilities to store milk [11]. Two recent systematic reviews found that interventions including dedicated legislation; support from family, employers and colleagues; access to convenient childcare; designated spaces for expressing milk; and flexibility to work from home were supportive of continued breastfeeding.

Focusing on the field of global health, we present two examples of barriers to breastfeeding that were particularly salient to us, as well as practical and creative solutions to addressing them.

Breastfeeding rooms/spaces: More workplaces are making breastfeeding rooms available, and they are a low-cost and proven intervention to support continued breastfeeding [12]. However, the mere designation of a room does not necessarily mean that it meets the needs of the workforce for whom it is created. For example, there might be physical barriers (long walk, security check, etc.) to access a room from one’s designated workspace. Creating more rooms isn’t always an option due to space and other resource limitations; furthermore, it is often difficult to find spaces that meet strict criteria to serve as a suitable breastfeeding room (e.g., running water, locking doors, refrigeration, sufficient electrical outlets, etc.). In many cases, restrooms become the default option, limiting their use for others, potentially introducing hazards, and risking contamination of pump parts that need to be assembled, used, and taken apart in an environment used primarily for human waste elimination. To overcome these challenges, managers and leaders can find creative solutions including calling a room something other than a formal ‘breastfeeding room’ so it can be set up more simply or making sure appropriate rooms are reserved for when breastfeeding mothers need them.

Meeting and travel planning: Meetings are a common part of our work, often requiring travelling significant distances and intensive hours or days engaging with partners and colleagues. Breastfeeding mothers know the experience of calculating the best time to dash out during a professional meeting while breasts uncomfortably swell with milk. When planning meetings, retreats and travel, managers should understand that there are specific windows during which nursing/pumping needs to occur and that a half hour or more time may be required to retrieve and assemble materials, pump, and then clean parts and safely store milk. Organizers should work to ensure that mothers can nurse/pump without missing key parts of the meeting, including meals, breaks and networking opportunities. Globally, breastfeeding rates of infants less than six months old have been shown to be 9% higher in countries were adequate breaks are mandated [13]. Further, organizers can ensure that anyone attending a meeting can access common nursing/pumping “first aid” items like access to clean, warm water, breast pads, and sanitizer, which could be bundled with other care-related items like medical kits, sanitary pads, masks, etc. This could be a required kit for all meetings that is used institution-wide and replenished regularly based on use.

Many solutions to mitigate barriers to breastfeeding or pumping need not be costly, nor do they require a complete upheaval of a workplace or professional activities. However, they do require awareness of the needs of those who are breastfeeding/pumping both within the workplace and while on duty travel or attending meetings and events. As we reflect on our own breastfeeding journeys, we are grateful for the people and workplace support measures that encouraged and enabled us and hopeful that our field can mitigate the numerous forms of exclusion we also faced.

The global health community is well-positioned to make breastfeeding more inclusive based on principles of equality, commitment to science, and fairness. However, as Rollins et al. noted in 2016, “Success in breastfeeding is not the sole responsibility of a woman — the promotion of breastfeeding is a collective societal responsibility” [14]. Extensive work is needed to address deep-rooted inequities [15]. We therefore urge global health organisations to review their current policies and infrastructure to more adequately support breastfeeding for those employees and other constituents who can and want to pursue it.

## Supporting information

S1 Table Detailed barriers to breastfeeding and actionable recommendations to overcome them. (DOCX)
