# Supplementary material for: Is breastfeeding ‘exclusive’? Barriers facing global health professionals and proposed solutions
Source: PLOS Glob Public Health. 2025 Mar 26;5(3):e0004377. doi: 10.1371/journal.pgph.0004377 (PMC11942364; doi:10.1371/journal.pgph.0004377)
Supplement: S1 Table — (DOCX) [file pgph.0004377.s001.docx]

**S1 Table: Barriers to breastfeeding and actionable recommendations to overcome them**

| 1. *Barriers and often-unseen burdens of breastfeeding while working in global health* | 1. *Measures to create an inclusive breastfeeding-friendly global health environment* |
| --- | --- |
| - Pumping breastmilk in unsanitary and non-private places like public toilets, cluttered admin offices, office kitchens, behind a screen while patients wait for care, in a parking lot or airplane seat, etc. - Figuring out how to safely store and transport breastmilk or alternatively dumping ‘hard-earned’ milk down the drain - Pumping in between meeting or conference sessions and missing out on networking and/or meal opportunities provided during those breaks - Falling behind on work due to time spent pumping, cleaning pump parts, negotiating milk storage, etc. - Managing clogged ducts, mastitis, and over- or under-production while away from baby - Building up an adequate supply of milk to leave for baby during work-related absences, or facing the feeling of failure for the inability to do so - Managing the grief and emotional load of missing one’s baby and not being able to nurse or directly feed while constantly pumping - Supporting the substantial cost of pumping equipment and/or formula because of reduced breastmilk production - Enduring late working hours leading to missed opportunities to breastfeed, and then requiring an extra pumping session when exhausted - Facing the promotion of breastmilk substitutes and ‘top feed’ that professional women are hounded by (especially in low- and middle-income countries) that militate against a commitment to exclusive breastfeeding - Costs to have your baby brought to you or accompany you for work meetings or work travel, including the cost of extra childcare, transport, and other logistics | *Organisational commitments:*   - Formal institutional support for breastfeeding, including breastfeeding leave (i.e. built-in breaktimes, decreased hours, flexible hours, or working from home arrangements) for at least one year, with room for reasonable flexibility and accommodations as determined by the mother - Adequate breaks and encouragement for mothers to schedule their time (i.e. block calendars, flexible working hours and protect breast/chest feeding or pumping sessions) - Organisational leadership and/or managers checking in with new mothers, especially as they transition back to work after maternity leave, to provide support and make clear it is not shameful or looked down upon to pump for or nurse your baby during the workday - Appropriate facilities for nursing and pumping in workplaces and at conference/ meeting venues that are conveniently located, clean, safe, and private - Workplaces that welcome options such as 1) bringing the child with a caregiver to facilitate breastfeeding the baby during the workday 2) providing onsite childcare options 3) allowing for partial remote work to ease the burden on working mothers.   *Work travel and meetings:*   - Line managers being flexible to accommodate breast pumping sessions when planning meetings - Childcare options provided at meetings and conferences, especially for children under two years of age. - Assistance locating local childcare for unavoidable work travel - When possible, offering flexibility with work travel during particularly the first year of a child’s life   *Peer support and awareness raising:*   - Breastfeeding awareness as part of training for all new staff but particularly those with management responsibilities, including the fact that not everyone is able to or chooses to breastfeed - An orientation to rights, facilities, and expectations for new mothers returning to work, including clear guidance on resources if they find themselves struggling - Creation of a peer group and/or support booklet to help new mothers navigate a particular workplace and support them to process their experiences and emotions as they return to the workforce |
